# Unit culture and respectful care in obstetric care settings: a qualitative concept elicitation study

**DOI:** 10.3389/fgwh.2026.1833740

**Published:** 2026-08-10

**Authors:** M. Takenoshita, S. L. Perez, A. J. Jethwa, R. Nath, E. K. Main, P. Sultan

**Affiliations:** 1 Department of Anesthesiology, Perioperative and Pain Medicine, Stanford University, Stanford, CA, United States; 2 Department of Public Health, California State University, Sacramento, CA, United States; 3 Department of Obstetrics and Gynecology, Stanford University, Stanford, CA, United States

**Keywords:** organizational culture, maternal health, respectful care, obstetrics, health inequalities

## Abstract

**Introduction:**

Unit culture and respectful care in hospital obstetric care settings are associated with patient outcomes and satisfaction. However, the definition and determinants of these concepts from the perspective of staff remain underexplored. We aimed to define unit culture and respectful care, identify their determinants and derive a conceptual framework describing their relationship in obstetric care settings.

**Methods:**

A multicenter exploratory qualitative study design was used. Following Institutional Review Board ethical approval, individual interviews were carried out with a volunteer sample of clinical and nonclinical staff working on labor and delivery, antepartum or postpartum units across California to capture comprehensive staff perspectives on obstetric care culture and respectful care. Participants were recruited between March to October 2025, selecting staff representing a range of maternal health providers and birth workers who served diverse regions, communities, and populations. The primary outcome was the definition and conceptual framework of unit staff culture and respectful care in obstetric settings.

**Results:**

Of the 22 participants who agreed to be interviewed, 21 completed interviews were included in the qualitative analysis (Asian 2/21, Black or African American 7/21, White 9/21, not disclosed 3/21). Participants included 9 physicians, 4 nurses, 4 doulas, 1 midwife, and 3 nonclinical staff across 16 healthcare organizations with equal representation of private, public and academic institutions. The mean duration of interview was 50 min. No new themes or subthemes were identified for three consecutive interview transcripts after 18 interviews, indicating thematic saturation had been achieved. Nine themes and 33 subthemes were identified. Unit culture was defined by teamwork, communication, and respectful treatment between staff. Respectful care was characterized by bias-free individualized care, and kindness and equity. Both concepts were influenced by leadership, psychological safety, and strategies to improve culture of respectful care, such as communication and training and staff education, as well as barriers to respectful care, such as resource constraints and implicit biases.

**Discussion:**

Unit culture and respectful care in obstetric settings were distinct and interrelated concepts. We present a conceptual framework identifying the modifiable determinants that shape both unit culture and respectful care, which can be used to inform improvement strategies.

## Introduction

Differences in organizational culture and respectful care contribute to inequities in maternal and infant health outcomes (1, 2). In the United States (US), Black women are three times more likely to die from pregnancy-related causes than White women (3). These disparities persist even after controlling for socioeconomic status and maternal clinical factors, suggesting that organizational determinants likely play a role (4). Unit culture and respectful care may present modifiable factors in improving inequities and advancing maternal health outcomes.

Both organizational culture and respectful care among staff and between staff and patients, are directly linked to patient outcomes and satisfaction, as well as staff well-being and performance (5, 6). For healthcare staff, negative culture and non-respectful care among staff are associated with worse quality of care, clinical performance, productivity, and patient outcomes and satisfaction (7). In contrast, positive organizational culture, characterized by effective staff communication, strong interpersonal relationships, and teamwork, is associated with improved patient safety and satisfaction (8–11), and greater work satisfaction, staff retention, and wellbeing (12–14).

Several features of obstetric care make it a particularly unique high-stakes environment to examine unit culture and respectful care. Obstetric scenarios can involve high stress, collaboration across multiple specialties, patients with multiple family member representatives, pre-conceived birth plans which may or may not be adhered to, unanticipated neonatal and maternal morbidity/mortality, known disparities in outcomes among underrepresented minority and lower socioeconomic groups, severe prolonged pain during labor, awake surgery and often rapidly changing and unpredictable scenarios, with severe consequences, requiring immediate assembly and close teamwork between diverse clinical and non-clinical staff. Obstetric staff face unique occupational stressors, such as adverse perinatal outcomes, including stillbirth and maternal death, which have considerable emotional implications. Taken together, these contextual characteristics and maternal health disparities highlight the need to evaluate unit culture and respectful care within obstetric settings.

Although respectful care in maternal health has been evaluated in studies (15), existing research has primarily focused on patient perspectives, and staff-to-patient interactions (15–17). While patient perspectives provide essential insights into experiences of care, it is also important to understand the environments and the factors that shape the environments in which patients receive care. Healthcare staff experience unit culture continuously, across multiple repeated interactions with leadership, colleagues and institutional systems, and are uniquely positioned to observe and influence organizational culture.

Moreover, studies of workplace culture and respectful interactions among healthcare staff have largely been conducted separately from investigations of patient experiences (18). This limits our understanding of how internally driven staff culture influences patient-facing care (18). Notably, prior reviews of patient-centered definitions of respectful care in maternity have not demonstrated clear benefit when applied in clinical quality improvement efforts (15). Therefore, understanding how diverse members of obstetric care teams define and experience respectful care is an essential step in identifying opportunities to improve respectful care for both staff and patients. Concept elicitation, a qualitative research process used to understand and define concepts, is a critical step to develop new measures capable of assessing relevant themes for quality improvement purposes (19). Concept elicitation in the maternal healthcare setting has led to the development of new measures for postpartum recovery (20, 21).

The primary aim of this concept elicitation study was to define unit culture and respectful care, identify their determinants, and derive an exploratory conceptual framework describing their relationship, in obstetric care settings.

## Materials and methods

An exploratory qualitative study design was used for this concept elicitation study. COnsolidated criteria for REporting Qualitative (COREQ) research reporting standards were followed (22). Individual interviews with clinical and nonclinical staff working on labor and delivery, antenatal or postpartum units, and neonatal intensive care units were carried out to capture comprehensive perspectives on unit culture and respectful care. Eligible participants were recruited between March to October 2025 following Institutional Review Board ethical approval from Stanford University (IRB 76075). The authors (MT, SLP, AJJ, RN, EKM, PS) engaged in reflexive practice throughout the study, starting with a discussion of their backgrounds, interests, and positionality on themes related to obstetric unit culture and respectful care prior to study commencement to identify and examine potential sources of bias that could influence the research process and interpretation of findings.

### Participants

Currently practicing healthcare professionals as well as nonclinical staff working on labor and delivery, antepartum or postpartum units were eligible for inclusion. These included, but were not limited to obstetricians, maternal-fetal medicine specialists, anesthesiologists, nurses, midwives, social workers, doulas, lactation consultants, administrators, clerical and custodial staff. Hospital leads from different hospitals and healthcare systems that have previously been involved in California Maternal Quality Care Collaborative (CMQCC) quality improvement programs were approached and asked if they were interested in having staff at their facility participate in the study. Those expressing interest were provided with recruitment flyers to share, in the form of an e-mail or a printout, with information for potential participants to contact the study team directly to express interest in participation. After informed consent was obtained, participants were invited to participate in individual semi-structured qualitative interviews. To capture a broad spectrum of healthcare and nonclinical staff perspectives, a purposive sampling method was used, selecting for participants that represented a range of maternal health providers and birth workers who served a range of diverse regions, communities, and populations across California.

### Data collection

Semi-structured interviews were carried out in English or Spanish by a bilingual research staff member with formal training in qualitative research methodologies (SLP) using an interview guide to provide a framework for discussion (Supplementary 1). The interview guide was developed in a similar manner to other maternal healthcare concept elicitation studies (21, 23), to facilitate a rich discussion on unit culture and respectful care in antenatal, postpartum and labor and delivery units, unit leadership and strategies employed by units to address culture and respectful care, observations of behavior between staff and between staff and patients, and experiences of disrespect. Demographic data, employment title, and the hospital of employment were collected. The anticipated duration of each interview was one hour. All interviews were conducted and digitally recorded over Zoom (San Jose, California), transcribed by a professional transcription service, de-identified, and translated into English for interviews conducted in Spanish. All participants were given a $75 gift voucher upon completion of the interview.

### Data analysis

Data was analyzed through inductive thematic analysis, using Braun and Clarke's six-phase framework (24), which involves familiarization with the data, generation of codes, and iterative identification, review, and definition of themes, and writing the report (24). The authors (MT, SLP, AJJ, RN, EKM, PS) independently reviewed two randomly selected transcripts, familiarized themselves with the data, and generated an initial set of codes without using a pre-existing conceptual framework. This enabled themes to emerge and inform the preliminary codebook. All 5 authors subsequently coded alternating transcripts from interviews with representatives from different hospital roles, iteratively refining the codebook following each transcript. Any discrepancies were discussed and resolved through consensus-building discussions following each transcript, until 100% agreement was achieved on code definitions and applications. The authors engaged in reflexive discussions throughout this iterative process (25). Coding was performed using the comments function in Microsoft Word (version 2510, Redmond, WA) (26). The process was repeated 21 times, until no further refinement of the code definitions was required and no new codes emerged for three consecutive transcripts, suggesting that theoretical saturation had been achieved (27). Eighteen versions of the codebook were developed during this iterative thematic coding process. The methodology is described in further detail in Supplementary 1.

The final codebook was then imported into NVivo (v.14, Lumivero, Denver, CO, USA). Intercoder reliability was assessed by calculating percent agreement between four coders (MT, SLP, AJJ, RN) among 4 independently coded transcripts using the final codebook. The 4 transcripts were selected as they represented a broad range of hospital staff roles and demographics. Analysis demonstrated 83.3% agreement, above the ≥80% threshold regarded as an acceptable intercoder reliability according to published standards (28). All of the transcripts were then coded by four authors (MT, SLP, AJJ, RN) in NVivo using the final codebook. Each transcript was coded by two of the four authors, and any discrepancies between the codes were discussed and resolved among the analysts.

### Study outcomes

The primary outcome of this study was to define unit culture and respectful care in obstetric care settings (antenatal, labor and delivery, and postpartum) and explore their determinants from the perspective of both clinical and non-clinical staff through identification of themes and subthemes related to these constructs, and to derive an exploratory conceptual framework to capture the relationship between these themes. Secondary outcomes included identification of themes and subthemes related to observations of behavior between staff and between staff and patients, experiences of disrespect, and the role of leadership.

### Qualitative analysis

Data analysis was performed using NVivo. Code definitions were adjusted for clarity and consistency, and codes were grouped into themes based on conceptual similarities and the prevalence of codes.

The sample size was estimated based on the anticipated number of participants necessary to achieve thematic saturation, when no new codes emerge from three consecutive interviews. Previous studies have suggested that thematic saturation is often achieved within 12 interviews (29). However, given the breadth of roles, professional and sociodemographic backgrounds that exist among staff in hospital settings, we anticipated that up to 20 participants may be required to capture staff experiences and perspectives. Data collection and analysis was carried out in parallel, and recruitment continued until thematic saturation was achieved.

## Results

Of the 22 participants who agreed to be interviewed, 21 completed interviews were included (95%) in the final qualitative analysis, representing broad sociodemographic and professional backgrounds across 16 diverse healthcare organizations. Participant and employer characteristics are summarized in Supplementary 2. The mean duration of interview was 50 min and the total interview time was 23 h 9 min; with 102,732 words analyzed.

**Figure 1:**
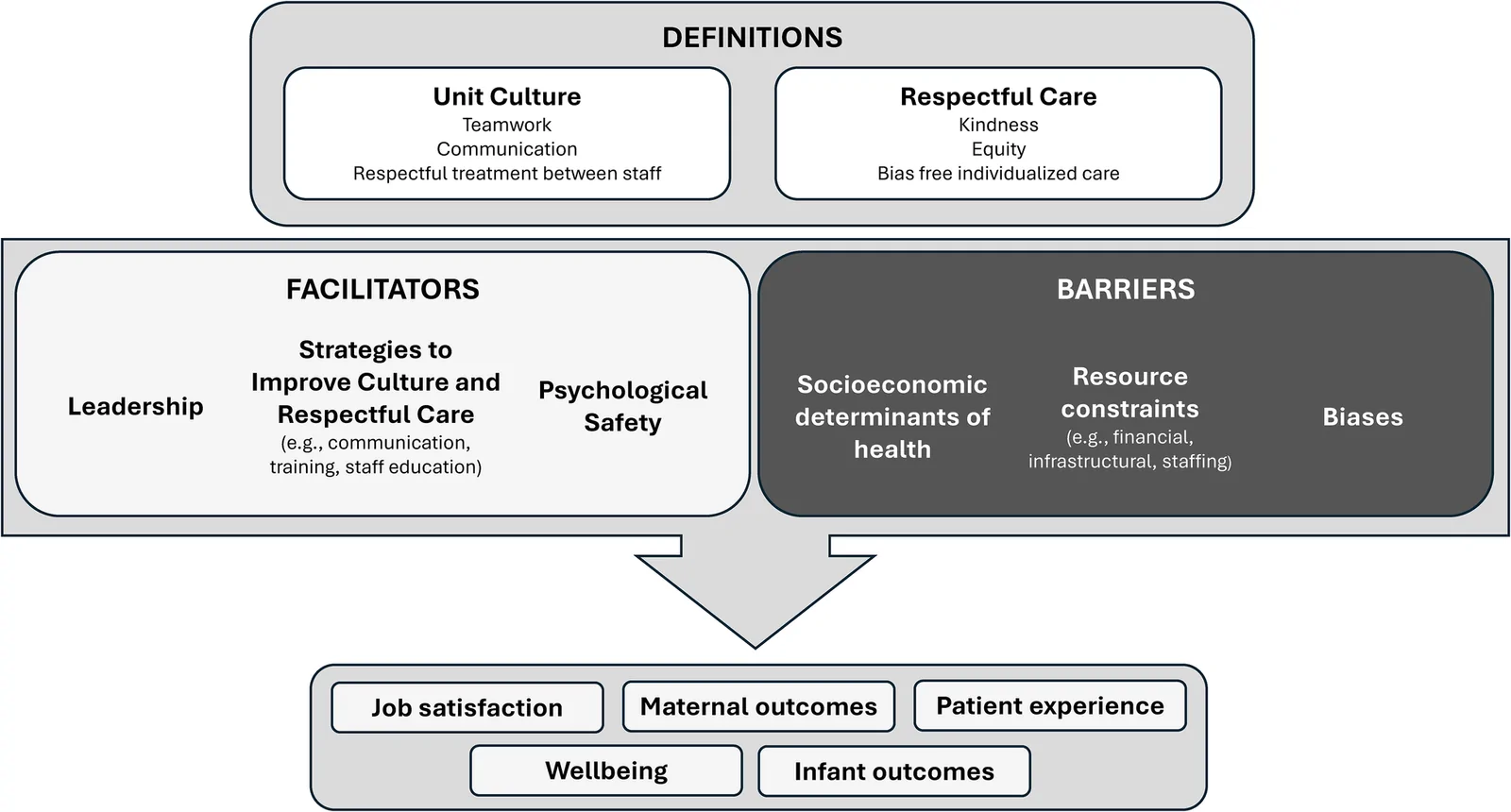
Conceptual framework of unit culture and respectful care, and their determinants. Facilitators to positive unit culture and respectful care include leadership, strategies to improve culture and respectful care, and psychological safety. Barriers include socioeconomic determinants of health, resource constraints, and biases. These impact staff job satisfaction and wellbeing, as well as maternal and infant outcomes and experiences.

Nine themes and 33 subthemes related to unit culture and respectful care in the context of obstetric care settings were identified (Table 1). A conceptual framework for staff culture and respectful care in obstetric care settings is provided in Figure 1.

**Table 1:** Summary of themes of staff culture of respectful care in obstetric settings.

**Theme**	**Subthemes**	**Summary**
Unit culture definitions and descriptions	Definition of unit culture relevant to hospital unit, positive descriptions of obstetric unit culture, negative descriptions of obstetric unit culture	Interviewees commonly referred to teamwork, shared understanding between colleagues, communication, and treating people with respect. Positive descriptions included strong collaboration across roles, equitable treatment, open dialogue, and a supportive learning environment. Negative descriptions commonly related to interprofessional conflict across roles and poor communication.
Respectful care definitions and descriptions	Definition of respectful care relevant to obstetric unit, examples of respectful care (staff vs. staff), examples of respectful care (staff vs. patients)	Respectful care was defined as “equitable” (8/17 participants) or “equal” (6/17 participants), bias-free care based on clinical need, characterized by respect, teamwork, kindness, and consistent standards. Common terms used to define respectful care were equity, patients, culture, everyone, people.
Non-respectful care	Characteristics influencing non-respectful care, examples of non-respectful care (between staff), examples of non-respectful care (from staff towards patients), examples of non-respectful care (from patients towards staff), impact of non-respectful care on patient treatment and care, impact of non-respectful care on staff treatment	Examples of non-respectful care from staff towards patients were reported more frequently than examples between staff members. Characteristics that influenced non-respectful care included sociodemographic factors (e.g., race, sex), professional factors (e.g., seniority, hierarchy between specialties) and clinical factors (e.g., substance misuse, obesity).
Strategies impacting respectful care	Onboarding processes/information, training and education, leadership strategies, communication strategies, encourage self-awareness, hospital strategies to improve respectful care and culture, identifying social needs of patients	Communication between staff, between staff and patients, and between hospitals and communities was considered central. Training strategies included formal education (e.g., online modules, didactics) as well as informal training delivered through conversations between staff members.
Assessment metrics	Assessment / metrics of unit culture or respectful care	Assessment metrics used by hospital units to evaluate respectful care included clinical outcome measures (e.g., neonatal intensive care admission rates) as well as a survey tool on patient safety culture.
Role as Leader	Influence of formal leadership role in improving culture and respectful care, perceptions of leadership	Visible leadership support and prioritization, hiring policies, and clear accountability when behavior or quality falls short contributed to improved culture and respectful care. Leaders were perceived as responsible for modelling behavior, and exerting top-down influence on respectful care.
Psychological safety	By leaders for staff, by staff for staff, by staff for patients, advocacy of patients, advocacy of staff	Patient advocacy and psychological safety were reported as significant determinants of unit culture and respectful care. They facilitated discussion and critical self-reflection regarding respectful care, while promptly addressing observed negative behavior.
Challenges and barriers to achieve respectful care	Challenges/barriers to achieve respectful care and outcomes on the hospital unit, challenges of responding to non-respectful care	Challenges included biases, communication, resources, staffing and workforce capacity, and socioeconomic determinants of health. Participants also reflected on the challenges of responding to non-respectful care.
Wellbeing	Importance of self-care as staff member, examples of self-care by staff members, challenges of caring for oneself as hospital staff member, burnout, guilt	Participants acknowledged the importance of self-care, recognizing the risk of burnout, and acknowledged challenges of caring for oneself.

### Unit culture

Fifteen participants defined unit culture in their interviews. Unit culture was defined by participants as the interpersonal dynamics, attitudes, shared priorities, and collective atmosphere that characterize how staff interact with each other and with patients. The most commonly articulated theme was the way staff show respect, kindness, dignity, and caring in their actions toward one another and toward patients (13/15; 87%). As one participant reported, unit culture is: “the interpersonal dynamic within the team itself  …  how we show up to work every day, how we interact with each other as coworkers, colleagues, peers, and how we convey mutual respect to each other within the team. And then as a result of those team dynamics, it contributes to the morale of the unit.” (Nurse). The atmosphere, or “morale” of the unit was mentioned by the majority (10/15; 67%) as a defining characteristic of unit culture. One participant described: “It's an energy, right? The energy that the unit has.” (Doula). Another reported: “it's the feeling when you walk into a unit, you know right away from conversations between the nurses, interactions between nursing and physicians, just listening to how they speak to the patients, what that culture is like. Is it cohesive? Is it compassionate? Is it volatile? Is it toxic?” (Nurse). A shared goal or mission was reported by almost half of the participants (8/15; 53%) as a core component of unit culture: “the kind of shared mission, drive, and philosophy of the unit and how people treat and interact with each other.” (Obstetrician). An equal number of participants (8/15; 53%) mentioned teamwork and collaboration across staff members as a defining feature. As one participant articulated, unit culture is “how the unit works collaboratively for successful outcomes.” (Obstetrician) The definitions of unit culture provided by the participants are described in greater detail in Supplementary 3.

### Respectful care

Seventeen participants reported on the definition of respectful care. Respectful care was characterized by bias-free individualized care, kindness and equity. The two most frequently reported defining features of respectful care were bias-free, need- or diagnosis-driven care (14/17; 82%), and individualized, person-centered care (14/17; 82%). As one participant described, respectful care was defined as: “Patients receiving care that is dictated by their level of clinical need and that is devoid of partiality or disparity or lack of care based on ethnicity, age maybe, and gender identification.” (Obstetrician) Respect, dignity, kindness, and humanization were described by the majority of participants (12/17; 71%) as a core component of respectful care. As described by participants, respectful care entails “exercising empathy to understand each individual's needs so that way we can then achieve the best possible outcome” (Nurse), and that “every patient would be treated with respect, would be heard, every family member.” (Nurse) Team-based and family-inclusive care was described by a majority of participants (9/17; 53%): “a very cohesive type of team, including the patient and the family.” (Nurse) The definitions of respectful care provided by the participants are described in greater detail in Supplementary 3.

### Determinants of unit culture and respectful care

Unit culture and respectful care were both influenced by psychological safety, leadership, and strategies to improve respectful care, such as communication and training and staff education, as well as barriers to respectful care, such as resource constraints and implicit biases.

### Obstetric context

While the themes of unit culture and respectful care, such as teamwork, communication and bias-free care, are broadly applicable across different healthcare settings, their examples illustrated the unique way that these factors manifest within the obstetric setting. For example, “non-respectful care,” the most frequently discussed theme, was most frequently related to race (11/21; 52% of participants, non-respectful care from staff towards patients, and 9/21; 43% non-respectful care between staff), with participants describing differential treatment unique to obstetric care. One participant recalled overhearing “Oh, that race or that particular last name means that they probably can't have a vaginal delivery, or it'll be really challenging, or pain control won't be good.” (Obstetrician) Other characteristics, such as drug and substance misuse (7/21; 33%) and weight and obesity (6/21; 29%) which have distinct implications in obstetric care, were notable drivers of non-respectful care from staff towards patients (Table 2, Supplementary 4): “for substance abuse, there's moms that are still using … but that's only maybe one in five … but they kind of just put all these moms of substance use in a bucket and treat them like they're not safe, and they're still using, and so I don't want them touching this baby.” (Nurse) Specific stigmatized clinical scenarios, such as termination of pregnancy, can lead to differential treatment: “We had a patient who had a nonviable baby … a few nurses had said,” “Oh, I don't want to care for her. Basically, it's an abortion.” (Nurse) The impact of the unit culture and non-respectful care on patients and their expectation from obstetric units is captured by the observation that “‘I want to make sure that I don't experience harm.’ … was a bullet point on a birth plan.” (Nurse) Interprofessional and intra-professional hierarchy between members of the obstetric unit were prominent sources of non-respectful care between staff members (11/21; 52% of participants), with one doula reporting: “I've had OBs who've said out of their mouth that doulas are just glorified back-rubbers.” (Doula) While the broad themes of respectful care and unit culture are broadly applicable across different settings, their triggers and manifestations were distinctly shaped by the obstetric context.

**Table 2:** Examples of non-respectful care and illustrative quotes.

Examples of non-respectful care	Characteristic	Number of interviewees N (%)	Illustrative quotes
From staff towards patients	Racism	11 (52)	"My white clients never, never encounter the line of questioning about fear of your partner, and are you safe at home…it doesn't happen.” (Doula)
	Drug and substance misuse	7 (33)	“I've heard doctors and nurses say inappropriate things about that parent for being in that situation. They treat them differently.” (Nurse)
	Weight/obesity	6 (29)	“I have just heard some comments that are just not clinical. And luckily, they're not in front of the patients, but I think they could be upsetting to staff or to the patient if they heard that.” (Anesthesiologist)
	Socioeconomic status/affluence	5 (24)	"But I had one client who showed up, again, low socioeconomic, all these things. And she told the nurse she had a doula. And she said, “You have a doula? How do you have a doula?"“ (Doula)
	Stigmatized medical illness	5 (24)	"Mental health…people talk about, “This mom has depression or postpartum depression,” … And I'm like, “Why are you talking here in the room, and the mom can hear you talking about her?”… It's not like you need to tell people's business in a room full of other people.” (Music Therapist)
	Staffing Levels	4 (19)	"I've worked other units where the parents are like, “I haven't had an update in the doctor in like three days.” And then you talk to the doctor, and they're like, “Yeah, I'm busy.”” (Nurse)
	Ageism	3 (14)	“[the physician] said to her, “Well, don't get mad at me if it hurts.” I mean, it got really personal. … So she ended up taking her mom with her, and the doctor did a 180. And part of it was because she looked really young.” (Doula)
	Hierarchical (specialty/ profession)	2 (10)	“those moms are the ones that really– like, “I wish I knew that I could have waited. I wish I knew that I didn't have to go ahead and get induced. But the doctor said this,” or, “They said that.” But I feel like they're not giving them conversations where they know that this is something that they should be able to decide together.” (Doula)
	LGBTQ+	2 (10)	“ I cared for a transgender patient, and it is something that I don't believe in… But I still stepped in and was very honest, like, “I may not call you a he. It will be very hard for me."“ (Nurse)
	Language barrier	2 (10)	“ virtual interpreters…. there's barriers to the technology. And then do you have enough of the technology available to support the number of patients on your unit at any given time where English is a second language?” (Nurse)
	Disability (physical/ mental/behavioral)	2 (10)	"I mean, I was at another hospital where the psychiatry department was essentially useless. They were basically there to say, “Do we 5150 of them or not?” Right? And if you're not 5150, they were just useless. “ (Obstetrician)
	Abortion	2 (10)	“we had a patient who had a nonviable baby…And a few nurses had said, “Oh, I don't want to care for her. Basically, it's an abortion."“ (Nurse)
	Hierarchical (seniority)	1 (5)	"she went to the client and said, “Are you sure that they can provide good support for you? Because after all, the program's free. The program is free to the client…I mean, do you really trust that that doula can really do this?” (Doula)
Between staff	Hierarchical (seniority)	11 (52)	"there was a lot of, “I've earned this place. You are the little one.” (Midwife)
	Hierarchical (specialty/profession)	11 (52)	"I've seen staff know that their job is to be quiet.” (Doula)
	Racism	9 (43)	"And it felt like it was a barrier because I can almost guarantee if I'd been White, blonde hair and blue-eyed, I don't think I would have had to be challenged the same way.” (Doula)
	Sexism	4 (19)	“you definitely sometimes see experiences where team members respond differently to male colleagues. I think every female physician has been called nurse repeatedly.” (Obstetrician)
	LGBTQ+	2 (10)	"resident who is in a same-sex relationship was sort of not harassed, but was talked to in a way that made her feel really uncomfortable about how a nurse was treating her based on her personal life” (Obstetrician)
	Staffing levels	1 (5)	"I've witnessed microaggressions in the sense of the provider gets angry because something is not going fast enough… but the unit's busy. We don't have enough staff” (Nurse)
From patients towards staff	Racism	1 (5)	“I had a anurse that was from Idia, and the patient says, ‘I don't like how she smells, and I need a new nurse’” (Nurse)

### Frequency of discussion

The 3 themes discussed by the highest proportion of interviewees were “respectful care descriptions and definitions,” “strategies impacting respectful care,” and “non-respectful care” (Table 3). The 3 most frequently described themes (total number of mentions from all interviews) were “non-respectful care,” “challenges and barriers impacting respectful care,” and “strategies impacting respectful care”. The proportion of participants discussing each theme and subtheme are summarized in Table 3, and exemplary quotes for each theme are illustrated in Table 4.

**Table 3:** Frequency of discussion of subthemes of unit culture and respectful care.

**Theme**	**Subtheme**	**Number of interviewees *N* (%)**
Role as leader	Influence of formal leadership role in improving culture and respectful care	11 (52)
	Perceptions of leadership	10 (48)
Psychological safety	By leaders for staff	6 (29)
	By staff for staff	3 (14)
	By staff for patients	6 (29)
	Advocacy of patients	13 (62)
	Advocacy of staff*	0
Unit culture descriptions and definitions	Definition of unit culture relevant to hospital unit	15 (71)
	Positive descriptions of hospital unit culture	9 (43)
	Negative descriptions of hospital unit culture	6 (29)
Respectful care descriptions and definitions	Definition of respectful care relevant to hospital unit	17 (81)
	Examples of respectful care (staff vs. staff)	11 (52)
	Examples of respectful care (staff vs. patients)	17 (81)
Assessment metrics	Assessment/metrics of culture or respectful care	8 (38)
Non-respectful care	Characteristics influencing non-respectful care	12 (57)
	Examples of non-respectful care (between staff)	17 (81)
	Examples of non-respectful care (from staff towards patients)	18 (86)
	Examples of non-respectful care (from patients towards staff)	2 (10)
	Impact of non-respectful care on patient treatment and care	9 (43)
	Impact of non-respectful care on staff treatment	1 (5)
Strategies impacting respectful care	Communication strategies	17 (81)
	Training and education	17 (81)
	Encourage Self Awareness	17 (81)
	Hospital Strategies to improve respectful care and culture	12 (57)
	Identifying social needs of patients	12 (57)
	Leadership strategies	11 (52)
	Onboarding processes/information	13 (62)
Challenges and Barriers impacting respectful care	Challenges/barriers to achieve respectful care and outcomes on the hospital unit	17 (81)
	Challenges of responding to non-respectful care	4 (19)
Wellbeing	Importance of self-care as staff member	8 (38)
	Examples of self-care by staff members	19 (90)
	Challenges of caring for oneself as hospital staff member	9 (43)
	Burnout	5 (24)
	Guilt	2 (10)

* This concept was introduced by the core group after consideration of the subtheme, “advocacy of patients”.

**Table 4:** Themes and subthemes of unit culture and respectful care and illustrative quotes.

**Theme**	**Illustrative quotes**
Role as leader	“If hospital leadership doesn't believe in equity or feel strong in supporting it, then that's going to trickle down to the rest of their unit and the rest of the doctors and nurses. And it's going to be felt by patients.” (Obstetrician)
Psychological safety	“This is an open conversation. It's not pointing fingers. We're going to be able to say what made everyone feel and do things a certain way and then turn around and grow from it rather than hold grudges.” (Midwife)
	“I recently overheard someone say, ‘I don't want to voice myself out of the job.’” (Midwife)
Unit culture descriptions and definitions	“They don't act different. If they see me using– I'm a housekeeping, and they talk to me the same, like a person, like with someone else. I'm in the labor and delivery to help the patients too because, in the role, I think it's the same. And doctors, nurse, and all the staff, we communicating a lot nicely. And I think it's– and I think, like I said, it's like a family over there.” (Housekeeping)
	“I mean, when we're talking negative energy, it's not really speaking to you but speaking at you. Maybe not having eye contact, or maybe carrying on and talking about something that's unrelated to my client.” (Doula)
Respectful care descriptions and definitions	“Patients receiving care that is dictated by their level of clinical need and that is devoid of partiality or disparity or lack of care based on ethnicity, age maybe, and gender identification. So all patients being treated in a way that optimizes and that prioritizes their needs without penalty for being different.” (Obstetrician).
Non-respectful care	“What makes me sad about birth plans is that the patient feels like they have to say, ‘I want to make sure that I don't experience harm.’ That was a bullet point on a birth plan.” (Midwife)
	“I've had OBs who've said out of their mouth that doulas are just glorified back-rubbers.” (Doula)
Assessment metrics	“Well, patient safety, there's perinatal core metrics that you can evaluate. That's severe maternal morbidity, maternal mortality, unexpected newborn complications, your normal spontaneous term vaginal delivery rates … So patient safety is really outcome-driven. What are the outcomes across the spectrum of care?” (Obstetrician)
Strategies impacting respectful care	“Everyone at our institution has to take those online forums or whatever where you kind of click through the modules … But that just isn't sufficient enough … We had a lot of really good luck in the past doing similar sort of small group discussions … patients coming and discussing their experiences.” (Obstetrician)
	“teaching self-awareness and empathy so that way you can drive positive culture as a leader is really difficult because sometimes when you're trying to create self-awareness for an individual, it requires disciplinary action, and that is hard as a leader.” (Nurse)
Challenges and Barriers impacting respectful care	“I just don't think that there's enough education or discussion about it … Anybody who discusses about it becomes offended with it.” (Nurse)
	“I would say probably the biggest are the social determinants of health … there are definitely many more barriers than if I was taking care of a population that was from a higher socioeconomic community, whether that's transportation, food, education.” (Obstetrician)
Wellbeing	“Taking care of the staff there at the bedside, also their mental health, I think, is really important, and allowing them time for that. And that really depends on where you work and the resources available.” (Nurse)
	“I quit clinical care. That's part of how I processed it” (Obstetrician)

The subthemes discussed in the highest proportion of interviews were “examples of self-care by staff members” and “examples of non-respectful care (from staff towards patients).” The most frequently reported subthemes (total mentions from all interviews) were “challenges/barriers to achieve respectful care and outcomes on the hospital unit,” “examples of non-respectful care (from staff towards patients),” and “communication strategies.” Illustrative quotes of examples of non-respectful care among staff and between staff and patients are summarized in Table 2. The final codebook and exemplary quotes for each subtheme are provided in Supplementary 4 and 5 respectively.

## Discussion

We inductively defined two distinct and interrelated concepts, unit culture and respectful care, and the factors that influence both in the context of obstetric settings. The two constructs were intimately linked: participants described how unit culture can both facilitate and hinder the delivery of respectful care, and respectful care between staff and staff and patients can shape unit culture. Both were influenced by leadership, psychological safety, and resource limitations.

The definition of unit culture presented in this study is consistent with key elements of established frameworks of organizational culture (30). Schein's (2010) model includes three levels of organizational culture: “artifacts”, which represent the observable features of culture; “beliefs and values” reported by the members as core to the organization; and basic “underlying assumptions”, the unconscious beliefs that ultimately drive behavior and practice (31). Applying this framework to our findings, the teamwork, respectful interpersonal treatment, and communication used by obstetric staff to define unit culture correspond to Schein's “artifacts”: the observable expressions of culture. They also described the values underlying these behaviors, such as the “shared mission, drive, and philosophy of the unit,” which represent Schein's proposed “beliefs and values” of culture. The “feel” or atmosphere of the unit described as a defining feature of culture reflect the “underlying assumptions”: the shared, unarticulated understanding of how the unit works, and staff are expected to behave.

Although the overarching concepts identified in this study, such as teamwork, communication, respect, and equity, are not unique to obstetric care, this does not detract from their relevance to the obstetric setting. Our inductive analytic approach was designed to allow concepts to emerge from participants' accounts rather than impose *a priori* assumptions that the resulting themes should be obstetric-specific. All themes were derived from the experiences and narratives of staff working within obstetric care settings, and their meaning was shaped by this context. Indeed, broad constructs such as respect and organizational culture may reasonably transcend individual clinical specialties; requiring such constructs to be unique to obstetrics could obscure concepts that are fundamental to healthcare interactions more generally. The obstetric specificity of our findings therefore lies not necessarily in the labels assigned to the overarching constructs, but in how they are experienced, triggered, and expressed within obstetric care settings. As illustrated in our findings, these manifestations included tensions surrounding birth plans, interprofessional relationships among obstetricians, nurses, midwives and doulas, stress associated with caring for two patients instead of one, stigmatization related to pregnancy loss and substance use, and assumptions regarding mode of birth and pain management. Thus, our findings suggest that broadly relevant constructs acquire distinct meaning and expression within the particular clinical, interpersonal, and organizational context of obstetric care. It is likely that many of the themes associated with respectful care and culture among staff members in obstetric settings are also evident on other medical wards in the hospital as the general framework for considerations for patient care are ubiquitous i.e., beneficence, non-maleficence, autonomy and justice, and therefore the staff interactions can overlap across specialties even after correcting for nuances in pathology and procedures performed in each specialty.

The definition of respectful care presented by our participants were consistent with existing frameworks of respect and disrespect in maternal healthcare (15–17). Cantor and colleagues summarized existing frameworks of respectful maternity care (RMC) into two different overlapping approaches: the absence of “disrespect and abuse,” such as physical and sexual abuse, non dignified care and stigma or discrimination (16, 17), and the application of “rights-based” “respectful care,” defined by the WHO as “organized for and provided to all women in a manner that maintains their dignity, privacy, and confidentiality, ensures freedom from harm and mistreatment, and enables informed choice and continuous support” (15, 32). Across the existing “rights based” RMC frameworks identified by Cantor and colleagues, dignity, empathy, equity, and autonomy are consistent themes (15). However, these existing frameworks have primarily focused on patient perspectives and patient-facing care (15–17). Our findings provide an additional perspective on these existing concepts by offering definitions derived directly from staff perspectives rather than those of patients.

Characteristics of non-respectful care identified by our participants are consistent with prior literature on disrespect in obstetric care settings. Racism was the most frequently described characteristic influencing non-respectful care both from staff towards patients (52% of participants) and between staff members (43%) among our participants. Similarly, race has been consistently associated with increased likelihood of non-respectful care in multiple healthcare settings, highlighting potential generalizability of our findings beyond labor and delivery units (33, 34). Our findings that staff themselves recognize and report racial bias as a common feature in obstetric care corroborate evidence that racial bias impacts differential treatment decisions and communication between staff and patients (35, 36). Moreover, racial discrimination is associated with adverse obstetric outcomes (37), and racial discrimination between staff members are likewise reported widely (38).

Interprofessional hierarchy was a prominent driver of non-respectful behavior among obstetric staff both between different professions, and between different levels of seniority within professions (52% of participants respectively). Lyndon and colleagues similarly report that communication failures in obstetric units are driven partly by hierarchical dynamics and psychological safety (11), and directly contribute to events that result in perinatal harm (11, 39). Consistent with this, medical hierarchy has been identified as a key driver of non-respectful treatment between staff (40–42).

### Determinants and a proposed conceptual model

Participants identified leadership, psychological safety and targeted strategies, including training and education, as determinants of unit culture in obstetric settings. Our findings are consistent with prior literature of culture in healthcare settings: collaboration, communication, leadership, and education, have been described as determinants of unit culture in non-obstetric settings (5, 6, 43–45). Similarly, other determinants of respectful care, such as the institutional “resources” (46, 47), accountability and transparency (15, 48), have been described in existing frameworks of RMC, which directly corroborate with our findings: “resources” (financial, 19%; infrastructural, 48%; workforce, 29%), “assessment metrics” (38%) and accountability (24%) were frequently described determinants of unit culture and respectful care.

Notably, there was considerable overlap in the determinants of respectful care and unit culture identified by the participants. These factors, including leadership, training and accountability practices, are directly modifiable. This is consistent with Schneider's definition of “organizational climate” a term used to describe the staff's shared perceptions of policies, reward structures and practices within the work environment (29). Organizational “culture” and “climate” are two distinct but related constructs: culture is deeper and slower-moving, while climate is more immediate and can be directly shaped by organizational interventions (30).

### Clinical and research implications

Unit culture and respectful care should be considered as key quality improvement indicators to advance both patient and staff outcomes (4). This approach parallels the use of standardized culture of safety assessment tools, such as the “Safety Attitudes Questionnaire,” which are used to identify opportunities to improve culture of patient safety in healthcare settings (49, 50). Although several survey tools exist to measure respectful maternity care from the patient's perspective, none have been specifically developed and validated for use among staff in obstetric care settings. The conceptual framework presented in this study may also inform the development of staff-focused standardized assessment of climate for respectful care, helping organizations to identify specific modifiable targets for quality improvement and monitor change over time. Further research is needed to validate this framework in obstetric settings beyond California, to identify the most effective strategies to improve staff culture of respectful care, and to examine the applicability and validity of this framework in other healthcare settings.

### Strengths and limitations

There are several limitations to this study. The volunteer sample of participants from California may be particularly motivated with respectful care initiatives and may not be representative of all healthcare workers involved in obstetric care, limiting external validity. The volunteers may have a particular interest in health equity, maternal health, or workforce management, or be motivated by particular experiences, positive or negative, that they have had in a personal or professional capacity. This may have influenced the opinions, experiences and perspectives disclosed in the qualitative interviews. Subcultures may also exist within and across hospital units, complicating efforts to capture a comprehensive understanding of unit culture. We aimed to address this by continuing recruitment and analysis until thematic saturation was achieved. Selection bias could be further minimized in future studies by replacing the open volunteer-only approach with stratified purposive maximum-variation sampling. However non-response and compliance may limit the utility of this approach.

## Conclusion

Obstetric culture was defined by teamwork, communication, and respectful treatment among staff. Respectful care was characterized by bias-free individualized care, kindness, and equity. This model can be used to guide discussions, develop measures for assessing climate for respectful care and inform strategies to improve outcomes in obstetric care.

## Data Availability

Data is available on reasonable request to the corresponding author.
