# Supplementary material for: Unit culture and respectful care in obstetric care settings: a qualitative concept elicitation study
Source: Front Glob Womens Health. 2026 Aug 10;7:1833740. doi: 10.3389/fgwh.2026.1833740 (PMC13502073; doi:10.3389/fgwh.2026.1833740)
Supplement: Supplementary file 1 [file Supplementaryfile1.docx]

Supplement 1. Methodology

**Supplement 1. Methodology – expanded**

**Reflexivity**

Reflexivity: The authors include researchers with experience studying maternal health, as well as a practicing obstetrician and anesthesiologist with ≥10 years experience, practicing in California. They represent diverse professional and personal backgrounds that may impact analysis and interpretation of data. All authors are committed to health equity, respectful maternal care that is sensitive to socioeconomic, cultural, demographic and clinical preferences of patients. They started this study with the assumption that implicit biases are prevalent, and impact behavior and care between staff as well as staff and patients. The authors were mindful of the impact that their experience, perspectives and positionality may have on the analysis and interpretation of data. In order to mitigate the influence of any personal biases on study findings, the authors engaged in reflexive practices, including team discussions of personal biases and its implications for data analysis and interpretation after each transcript.[1]

**Sample size**

Sample size: The number of participants required to complete this study was determined based on the anticipated number necessary to achieve thematic saturation.[2–4] According to previously published studies, saturation was defined as when no new themes emerge across three consecutive interviews.[4] Previous research indicates that saturation is often reached within the first 12 interviews, with fundamental thematic elements sometimes appearing as early as six interviews.[5] Given the diversity of roles across multiple institutions that we planned to include in interviews, we aimed to interview a minimum of 20 participants but planned to interview more if needed to achieve thematic saturation. Based on our previous concept elicitation work surrounding complex multidimensional constructs[6–8] It was felt that this number of participants would likely achieve thematic saturation across staff roles in geographically diverse obstetric settings in California.

1.	Olmos-Vega FM, Stalmeijer RE, Varpio L, Kahlke R (2023) A practical guide to reflexivity in qualitative research: AMEE Guide No. 149. Med Teach 45:241–251. https://doi.org/10.1080/0142159X.2022.2057287

2.	Bowen GA (2008) Naturalistic inquiry and the saturation concept: a research note. Qualitative Research 8:137–152. https://doi.org/10.1177/1468794107085301

3.	Saunders B, Sim J, Kingstone T, et al (2018) Saturation in qualitative research: exploring its conceptualization and operationalization. Qual Quant 52:1893–1907. https://doi.org/10.1007/s11135-017-0574-8

4.	Francis JJ, Johnston M, Robertson C, et al (2010) What is an adequate sample size? Operationalising data saturation for theory-based interview studies. Psychol Health 25:1229–1245. https://doi.org/10.1080/08870440903194015

5.	Guest G, Namey E, Chen M (2020) A simple method to assess and report thematic saturation in qualitative research. PLoS One 15:e0232076. https://doi.org/10.1371/journal.pone.0232076

6.	Sultan P, Jensen S, Taylor J, et al (2022) Proposed domains for assessing postpartum recovery: a concept elicitation study. BJOG 129:9–20. https://doi.org/10.1111/1471-0528.16937

7.	Bekemeyer Z, Takenoshita M, Michel G, et al Proposed domains of postpartum sleep: a concept elicitation study using qualitative interviews. SLEEP Advances. https://doi.org/doi:10.1093/sleepadvances/zpaf068

8.	Callihan P, Cottrell Bekemeyer ZJ, Pedreira R, et al (2025) Intraoperative pain during cesarean delivery: a qualitative concept elicitation study. Int J Obstet Anesth 64:104748. https://doi.org/10.1016/j.ijoa.2025.104748

**Semi-structured interview procedure and interview guide**

**The interviewer**

The semi-structured interviews were conducted by a health services researcher (SLP) with formal training in qualitative research methodologies. With over 15 years of experience designing, conducting, and leading qualitative studies, with particular emphasis on maternal and reproductive health, this background informed the methodological rigor of the study and supported systematic, reflexive data collection and analysis.

SLP identifies as an Asian cisgender woman, which may have shaped both her interest in the research topic and her interactions with participants. Her professional background reflects a strong commitment to improving equity, access, and quality of care in maternal and reproductive health systems. She entered the research with the assumption that structural and systemic factors – such as healthcare access, policy environments, and social inequities – play a significant role in shaping reproductive health outcomes. While she sought to remain neutral and open during data collection, she acknowledges that her prior research experience and advocacy-oriented interests may have influenced the framing of interview questions and the interpretation of participants’ responses. Reflexive practices, including memo-writing and team discussions, were used to mitigate potential bias and enhance analytic rigor.

She had no prior relationship with the interviewees prior to study commencement. The interviewees were made aware of her occupation, and the aim of the study prior when participating in the study.

**Interview procedure**

Virtual interviews were conducted one-on-one between the participant and the researcher, using an interview guide. The semi-structured interview guide was developed to facilitate a rich discussion on staff culture of respectful care in antenatal and labor and delivery units, unit leadership and strategies employed by units to address culture of care, observations of behavior between staff and between staff and patients, and experiences of disrespect. The guide was reviewed by content and qualitative research experts, and questions were assessed for clarity, relevance, and interview duration. It was not formally pilot tested in its entirety. The interview guide is included in the following page. Participants were encouraged to join from a private, quiet location; however, the presence of others in participants’ settings could not be independently verified. Field notes were taken intermittently during and after interviews using handwritten notes.

**Interview guide**

**Interview start**

Thank you for making the time to meet with me. I’m Susan Perez and I’m here on behalf of Stanford University and the California Maternal Quality Care Collaborative (CMQCC). Today we’re going to be talking about hospital culture in the context of respectful care. What we learn from you today will inform the development of a Hospital Culture of Respectful Care survey. This focus group/interview will last about 2 hours/1 hour.

As a participant, I want to emphasize that you have permission to skip questions. You can simply say PASS when you don’t want to respond, you can also choose silence. Your participation is voluntary which means that at any moment you can choose if, when, and how you want to share or not share and/or you want to participate or not participate. Ahead of this discussion, we shared with you a consent form. Please let me know if you have any questions about what you read.

I want to let you know that I am recording this session so we can capture all the comments accurately. We will create transcripts that will not include anyone’s name. After the information is summarized, the recordings will be destroyed. Any questions so far?

1. I’d like to start with learning a bit more about your background. Would you please share:
   a. The racial and ethnic groups with which you identify.
   b. Your role within this hospital unit and the number of years you have been in the current role?

Throughout this interview we will be discussing unit culture and respectful care. There are a lot of different definitions for these terms.

1. How do you define or describe the term “unit culture”?
2. We know across the nation and in California, there are disparities in the treatment of patients and clinical outcomes due to differences with how patients are treated during labor and delivery. Throughout this conversation we will be diving deep into respectful care within hospital units.
   a. How would you define respectful care when it comes to patient treatment, care, and outcomes?
   b. What are important factors for you and your colleagues regarding respectful care in the workplace?
   c. What are the shared goals for promoting respectful and equitable care on your hospital unit?
      i. Respectful care between hospital staff?
      ii. Respectful care with patient treatment and care?

**[Artifacts** (Actions and representations visible for observers and openly communicated)]

***For interviews and focus groups: Remind the participants not to divulge any identifiable information about themselves or about others.**

1. Have you seen staff treat other staff differently based on differences, such as culture, race, age, training, seniority, or other factors?
   a. Please tell me more about this situation.
   b. Have you experienced different or harmful treatment from colleagues or other staff?
2. Have you seen staff treat patients differently based on the patient’s race, ethnicity, cultural or religious practices, age, weight, mental health history, substance use, or social economic status, or other factors?
   a. Please tell me more about this situation.
3. Tell me about the ways your hospital unit talks about inequities on your hospital unit. For example, with racial inequities are difficult to talk about. People often don’t have the words to have these conversations or feel defensive.
   a. What communication tools, if any, do you use to discuss issues of non-respectful care such as racism, harm, or disrespect toward patients?
      i. With each other?
      ii. As a unit?
   b. What determines if these issues are addressed?
      i. How does trust within your unit play a role in how these issues are addressed?
   c. Are there groups or subsets of workers that experience non-respectful care in the workplace?
   d. How do you think inequities impact staff? This can include inequitable treatment toward staff and when staff observe inequitable treatment of patients.
4. When it comes to addressing inequities, disrespectful care, or harm what learnings, technical assistance, or professional development are you engaging in as a unit?
   a. How is information shared or communicated?
   b. How do you hold each other accountable to best practices or applying what was learned?
   c. What is your perception of online modules/onboarding education about topics of inequities, disrespectful care, or harm to patients?

**[Beliefs and values** (Evaluations and judgments that are not visible but mostly accessible by questionnaires or other methods)]

1. What is the role of your hospital leadership in supporting or promoting respectful care?
   a. What is needed from hospital leadership to ensure respectful care is achieved? I encourage you to dream big with resources and money being no object.
2. When onboarding or orienting new staff into your department, what would you tell them about the culture of your unit?
   a. How would you describe the communication processes?
   b. How do you hold each other accountable to best practices?
   c. What happens when someone observes someone doing something wrong? Or there is an adverse event on the hospital unit?
      i. How do you support each other through difficult decisions or outcomes?
   d. What’s a tip you would give someone to quickly and smoothly jump-in as a member of the hospital unit?
      i. Why this tip?
3. Have you worked in other hospital labor and delivery units?
   a. [IF YES] What are some differences between the two unit’s hospital cultures?
   b. Have you worked on different shifts within this hospital unit?
      i. [IF YES] What are some differences between the two shift’s cultures?

**[Assumptions** (Deep formatting of the mind, such as values that are so internalized in individuals and organizations that they are taken for granted.)]

1. It is well documented that microaggressions are common in hospitals. Microaggressions are actions, statements, and other behaviors—unintentional or intentional—that discriminate against people in marginalized communities. Microaggressions, in the form of statements or actions, can be made toward other staff or patients.
   a. Some examples include:
      i. During a code run by a female physician, the nurse asks what medications the male physician would like to order. The assumption is the physician who is in charge is most likely the male physician.
      ii. A Black patient complains of pain and a physician does not examine them and discharges them. The assumption is that Black patients can tolerate higher levels of pain.
   b. What are some examples of microaggressions you have witnessed?
   c. What are some examples of microaggressions you have experienced?
2. What are some examples of assumptions, good and bad, that you observe staff make about patients?
3. If respectful care meant there were *consistently* no disparities across all clinical outcomes.
   a. Tell me about things that would make it easy or hard to achieve respectful care and outcomes on your hospital unit.
   b. What are the individual responsibilities or efforts that need to happen to achieve this goal?
   c. What are the unit level actions and initiatives that need to take place to achieve this goal?
   d. What actions does the hospital need to take to achieve this goal?
   e. What assumptions and behaviors need to be questioned to achieve respectful care?
   f. If given all the resources and put in charge, what would you do first to achieve this goal within the next 5 years? I acknowledge this is likely an impossible goal, but we’re dreaming ambitiously.
      i. How do you envision your hospital unit team comes together to achieve this goal?
         1. In terms of teamwork, what is already in place to make this successful?
         2. In terms of teamwork, what needs to change to make this successful?
4. Recognizing the root of most inequities start outside the hospital, such as lack of social support or chronic conditions, what is being done to understand the communities, circumstances, and environments of the individuals who are coming to your hospital?
   a. What are examples of relationships you have established with community partners?
   b. What relationships does your hospital have with community resources to support patients’ transition to their community during the postpartum period?
      i. For example, if a patient was experiencing food insecurity, what is the process to ensuring they are food secure?
   c. What is being done to understand the living conditions and lived experiences of patients?
      i. What do you know about the conditions and circumstances they go home to or are coming from?
5. We discussed a lot of heavy topics today. Working in healthcare is not easy and we are seeing this in hospital staff burnout rates.
   a. How do you process traumatic situations or difficult days?
      i. Do you do this alone or process with others?
         1. Who are the people you might turn to after a difficult day at work?
   b. Could you provide an example?
6. Is there anything else you think I should know? Do you have any questions for me?

**End interview**

1.	Olmos-Vega FM, Stalmeijer RE, Varpio L, Kahlke R (2023) A practical guide to reflexivity in qualitative research: AMEE Guide No. 149. Med Teach 45:241–251. https://doi.org/10.1080/0142159X.2022.2057287

2.	Bowen GA (2008) Naturalistic inquiry and the saturation concept: a research note. Qualitative Research 8:137–152. https://doi.org/10.1177/1468794107085301

3.	Saunders B, Sim J, Kingstone T, et al (2018) Saturation in qualitative research: exploring its conceptualization and operationalization. Qual Quant 52:1893–1907. https://doi.org/10.1007/s11135-017-0574-8

4.	Francis JJ, Johnston M, Robertson C, et al (2010) What is an adequate sample size? Operationalising data saturation for theory-based interview studies. Psychol Health 25:1229–1245. https://doi.org/10.1080/08870440903194015

5.	Guest G, Namey E, Chen M (2020) A simple method to assess and report thematic saturation in qualitative research. PLoS One 15:e0232076. https://doi.org/10.1371/journal.pone.0232076

6.	Sultan P, Jensen S, Taylor J, et al (2022) Proposed domains for assessing postpartum recovery: a concept elicitation study. BJOG 129:9–20. https://doi.org/10.1111/1471-0528.16937

7.	Bekemeyer Z, Takenoshita M, Michel G, et al Proposed domains of postpartum sleep: a concept elicitation study using qualitative interviews. SLEEP Advances. https://doi.org/doi:10.1093/sleepadvances/zpaf068

8.	Callihan P, Cottrell Bekemeyer ZJ, Pedreira R, et al (2025) Intraoperative pain during cesarean delivery: a qualitative concept elicitation study. Int J Obstet Anesth 64:104748. https://doi.org/10.1016/j.ijoa.2025.104748
