# Supplementary material for: Unit culture and respectful care in obstetric care settings: a qualitative concept elicitation study
Source: Front Glob Womens Health. 2026 Aug 10;7:1833740. doi: 10.3389/fgwh.2026.1833740 (PMC13502073; doi:10.3389/fgwh.2026.1833740)
Supplement: Supplementary file 2 [file Supplementaryfile2.docx]

Supplement 2. Participant and employer characteristics

# 1 Supplementary Data

A total of 56 eligible participants across 29 healthcare institutions were approached. Twenty-two participants across 16 organizations were consented and recruited into the study. Of these, interviews from 21 participants from 16 different healthcare institutions were included in the qualitative analysis.

# 2 Participant characteristics

The 21 interviewees included in the analysis consisted of 9 physicians (Obstetrics and gynecology attending = 7, Anesthesiology attending = 1, resident physician for Obstetrics and gynecology = 1), 4 nurses, 1 midwife, 4 doulas, and 3 non-clinical staff (music therapist = 1, housekeeping =1, ward receptionist = 1). Of these, 19 were female and 2 were male, with broad racial (Asian = 2, Black or African American = 7, White = 9, chose not to disclose = 3) and ethnic representation (Hispanic = 6).

# 3 Employer characteristics

Participants represented diverse healthcare organizations. Geographically, 10 of the participants were recruited from organizations in Northern California, while 1 was recruited from Central California and 10 participants were employed by institutions in Southern California. Participants were recruited from urban (9/21 participants), suburban (7/21 participants), and community organizations (4/21 participants). Five of the institutions were academic centers, while 4 were community organizations, 6 were private, and 5 were public hospitals.
