# Supplementary material for: Unit culture and respectful care in obstetric care settings: a qualitative concept elicitation study
Source: Front Glob Womens Health. 2026 Aug 10;7:1833740. doi: 10.3389/fgwh.2026.1833740 (PMC13502073; doi:10.3389/fgwh.2026.1833740)
Supplement: Supplementary file 3 [file Supplementaryfile3.docx]

Supplement 3. Codebook

| **Domain** | **Subdomains** | **Definition** |
| --- | --- | --- |
| **Role as leader** | **Influence of formal leadership role in improving culture and respectful care** | How formal leadership role affects culture of the unit. E.g. Hiring practices. |
|  | **Perceptions of leadership** | What staff think of the leader, and what leaders think of themselves. |
| **Psychological safety** | **By leaders for staff** | Leaders facilitating or creating a safe space to speak out. |
|  | **By staff for staff** | Staff making staff feel safe. |
|  | **By staff for patients** | Staff making patients feel safe to speak out. |
|  | **Advocacy of patients** | Speaking up on behalf of a patient; includes staff role / in their job. |
|  | **Advocacy of staff** | Speaking up on behalf of another staff member; includes staff role / in their job. |
| **Unit culture descriptions and definitions** | **Definition of unit culture relevant to hospital unit** | Interviewee's definition of unit culture relevant to hospital unit. |
|  | **Positive descriptions of hospital unit culture** | Positive descriptions of hospital unit culture. |
|  | **Negative descriptions of hospital unit culture** | Negative descriptions of hospital unit culture. |
| **Respectful care descriptions and definitions** | **Definition of respectful care relevant to hospital unit** | Interviewee's definition of respectful care relevant to hospital unit. |
|  | **Examples of respectful care (staff vs staff)** | E.g. Empathetic care, trust, shared decision making; trust building. Not limited to first person/hand experience. |
|  | **Examples of respectful care (staff vs patients)** | Being respectful of different cultures e.g. Singing to children, having access to herbs / medications, culturally competent care. Not limited to first person/hand experience. |
| **Assessment metrics** | **Assessment / metrics of culture or respectful care** | Specific markers/measures e.g. Number of cases/percentages/safe reports/friends and family test; quality improvement dashboard. |
| **Non-respectful care** | **Characteristics influencing non-respectful care** | Characteristics that influence non-respectful care e.g., race, ethnicity, cultural or religious practices, age, weight, mental health history, substance use, or social economic status. |
|  | **Examples of non-respectful care (between staff)** | Staff factors include race/ethnicity, culture, age, training, sexual orientation, and seniority. Includes microaggressions, emotional and physical harassment, a specifically witnessed action or statement; as well as unprofessionalism, lack of professional courtesy, and lack of self-awareness or insight. Not limited to first person/hand experience. |
|  |  |  |
|  | **Examples of non-respectful care (from staff towards patients)** | Including microaggressions, emotional and physical harassment, specifically witnessed action or statement, as well as unprofessionalism, lack of empathy or patience. Microaggression is defined as an action or verbal comment which makes people feel uncomfortable; microaggression is defined by the subjective experience of the victim/recipient. Not limited to first person/hand experience. |
|  | **Examples of non-respectful care (from patients towards staff)** | From patients, directed towards a staff member. Including microaggressions, emotional and physical harassment, specifically witnessed action or statement, as well as unprofessionalism, lack of empathy or patience. Microaggression is defined as an action or verbal comment which makes people feel uncomfortable; microaggression is defined by the subjective experience of the victim/recipient. Not limited to first person/hand experience. |
|  |  |  |
|  |  |  |
|  | **Impact of non-respectful care on patient treatment and care** | Reaction, feeling secondary to example or incident. |
|  | **Impact of non-respectful care on staff treatment** | Reaction, feeling secondary to example or incident. |
| **Strategies impacting respectful care** | **Onboarding processes / information** | Onboarding training / tips for new hire. |
|  | **Training and education** | Can be positive or negative examples. E.g. Nurse training, including implicit bias training. |
|  | **Leadership strategies** | Team leader led initiative, action based, examples of open-door policy |
|  | **communication strategies** | How communication is used to achieve respectful care e.g. Multimedia, handouts. How information is communicated to patients or staff. Lack of patient complaints, how to stand up for oneself; negative descriptions of communication, intentional feedback to influence culture and behavior. |
|  | **Encourage Self Awareness** | How a system works for an individual or realizing inequities are an issue; improving respectful care through self-improvement; understanding risk/benefit ratio as a provider, including accountability (to staff, to leadership, to patients) |
|  | **Hospital Strategies to improve respectful care and culture** | Division or department-led initiative. E.g. commitment to equitable care documents; policies/protocols informed by data, e.g. Quality improvement meetings, health equity tasks; safe reports, quality reviews |
|  | **Identifying social needs of patients** | E.g. Partnering with communities, navigating social services |
| **Challenges and Barriers impacting respectful care** | **Challenges / barriers to achieve respectful care and outcomes on the hospital unit** | Includes changing population that is being served e.g. religion, ethnicity, e.g. Trying to start initiatives/making a change but facing bureaucratic challenges to execute this; resources e.g. Inadequate staff, also lack of trust |
|  |  |  |
|  | **Challenges of responding to non-respectful care** | Examples of how people find it difficult to respond to non-respectful care. |
| **Wellbeing** | **Importance of self-care as staff member** | Reflections on why self-care is needed, coping strategies. |
|  | **Examples of self-care by staff members** | What participants are doing for selfcare. |
|  | **Challenges of caring for oneself as hospital staff member** | Examples of challenges of caring for oneself as hospital staff member. |
|  | **Burnout** | Emotional, physical, mental sequelae e.g. Burden of taking responsibility in advocating for respectful care. |
|  | **Guilt** | Blaming oneself e.g. For not speaking up, having bias/prejudging a patient. |
