# Supplementary material for: Unit culture and respectful care in obstetric care settings: a qualitative concept elicitation study
Source: Front Glob Womens Health. 2026 Aug 10;7:1833740. doi: 10.3389/fgwh.2026.1833740 (PMC13502073; doi:10.3389/fgwh.2026.1833740)
Supplement: Supplementary file 4 [file Supplementaryfile4.docx]

Supplement 4. Domains and subdomains of staff culture of respectful care and illustrative quotes

| **Domain** | **Subdomains** | **Definition** | **Illustrative quotes** |
| --- | --- | --- | --- |
| **Role as leader** | **Influence of formal leadership role in improving culture and respectful care** | How formal leadership role affects culture of the unit. E.g. Hiring practices. | "I've actually created a birth preferences planning document to help normalize that all people want is to express their hopes and desires and to make sure that everyone understands that they want to just feel safe and aligned. And so instead of coming into it like, "Oh, this person's going to be difficult," that it really is just a place of just communication." (Midwife)  "it was finally recognized at the CEO level, and they let the people off that were causing those type of problems. And then they got new leaders, and it was nice to see the change and shift of the culture of the unit. So I think it goes all the way from the top, all the way to the bedside." (Nurse)  "At the department level, our department chair has also been very active in supporting DEI roles. We have a department DEI committee that also-- he just sort of gives us free reign on what we think we need to do. We have a community fund" (Obstetrics)  "I think it's very important because I feel everything stems from the top. So if the top doesn't view it as important, why would staff view it as important? It's kind of like a parent and a child. " (Nurse). |
|  | **Perceptions of leadership** | What staff think of the leader, and what leaders think of themselves. | "I have a position in which I am empowered to do that type of work and to bring those goals in" (Nurse)  "Leadership needs to understand their own biases. And they need to support a culture of being able to feel safe to be able to voice your concerns when these occur and then also supporting quality improvement initiatives for staff at the bedside to be a part of because then you really get buy-in from them and you really make that long-term effect." (Nurse)  "if hospital leadership doesn't believe in equity or feel strong in supporting it, then that's going to trickle down to the rest of their unit and the rest of the doctors and nurses. And it's going to be felt by patients. I think their role is engagement in those conversations, supporting curriculum and continued education" (Obstetrician) |
| **Psychological safety** | **By leaders for staff** | Leaders facilitating or creating a safe space to speak out. | "if there is something happening on the floor, on labor and delivery, and I'm not available to answer your question or your concerns, you don't need to go through me... You just go and talk to the attending. I kind of think of that as equity, too, everyone having a voice on my team, or feeling confident and safe to have a voice." (Obstetrician, Resident)  "you should be comfortable having a voice to say something and a pathway to do that." (Nurse) |
|  | **By staff for staff** | Staff making staff feel safe. | "I do think that people feel safe saying things, maybe too safe. So then maybe it causes a little bit of friction to each other. But at the same time, we're trying to grow and teach everyone emotional intelligence and being like, "This is an open conversation. It's not pointing fingers. We're going to be able to say what made everyone feel and do things a certain way and then turn around and grow from it rather than hold grudges," whether it's speaking up of either treating each other wrong or treating patients wrong, so." (Midwife) |
|  | **By staff for patients** | Staff making patients feel safe to speak out. | "And so one of the things we did after - actually, I caught a significant mess of a patient who'd had an eclamptic seizure during an attempted home birth - was to reach out to the home birth community to set up quarterly meetings led by a midwife, not led by a doctor, to basically lower the barrier to entry to say to everybody, "You can come here no matter what. We're not going to steal your patient. We're not going to badmouth you. We're not going to say, 'You shouldn't have sent her in.' " (Obstetrician) |
|  | **Advocacy of patients** | Speaking up on behalf of a patient; includes staff role / in their job. | "I personally will bring it up to my client in a way that it allows the doctor or the nurse to step back and think about what's going on and to communicate with them, so then they feel like they're having a choice in what's happening while they're in labor." (Doula)  "I make sure patients and our staff understand, as a midwife, our foundation is that we just honor every human for who they are. We want to meet them to their expectations by also guiding them to have the highest level of healthcare that they can receive and knowledge to advocate for themselves. And I think we're also a bridge to help everyone feel safe and understood." (Midwife)  “sometimes it does take individual healthcare providers to be the voice for the vulnerable patients and speak up for them because labor and delivery and NICU is their most vulnerable time, so." (Nurse) |
| **Unit culture descriptions and definitions** | **Definition of unit culture relevant to hospital unit** | Interviewee's definition of unit culture relevant to hospital unit. | "how well the unit functions as a team across disciplines, across genders to some extent, and across ethnicities and racial backgrounds...making sure that the patients get the care that they need. So optimizing the outcome, everybody with the shared goal of making sure that the best level of care is provided for the patient and her pregnancy, her fetus" (Obstetrician)  "It's how you relate to each other, how you relate to the patients, how you understand your boundaries, how you understand what you should normally do in a certain situation, right? So something's going on. Do you call the doc? Do you call the doc and a midwife? Do you call security if the patient wants to leave AMA? Do you let her leave AMA if she signs the paperwork? I mean, all of these little things are unit culture" (Obstetrician)  "It's an energy, right? The energy that the unit has. Or if I were to describe it, unit culture-- I never even thought about it like that. But yeah, I would say a way of being, different ways they treat people, how they talk to people, how it feels, things like that." (Doula) |
|  | **Positive descriptions of hospital unit culture** | Positive descriptions of hospital unit culture. | “some hospitals embrace the doula, and they see the doula part as a part of the treatment team. So there's open dialogue, or maybe I see something, I make a suggestion. They make a suggestion. We kind of collaborate and work together" They'll ask about you. "What do you need to be able to support the client? What things have you worked with with the client?" so that there's this sense of mutual respect." (Doula)  "They don't act different. If they see me using-- I'm a housekeeping, and they talk to me the same, like a person, like with someone else. I'm in the labor and delivery to help the patients too because, in the role, I think it's the same. And doctors, nurse, and all the staff, we communicating a lot nicely. And I think it's-- and I think, like I said, it's like a family over there." (Housekeeping)  "It's like they're so much more helpful. They are willing to go beyond what their role is without hesitation. Without-- I mean, it is different. It just feels so much more-- I mean, united more. Just gung-ho, "Let's do this. Let's get this day conquered." I mean, it's just more family-oriented, more friendly. " (Front desk) |
|  | **Negative descriptions of hospital unit culture** | Negative descriptions of hospital unit culture. | "You're perceived almost like a visitor." " It was not like, "Oh, you're a doula. Thank you for coming. We're excited. We want to support you." It was more of like, "Well, who are you?" (Doula)  "I mean, when we're talking negative energy, it's not really speaking to you but speaking at you. Maybe not having eye contact, or maybe carrying on and talking about something that's unrelated to my client. " (Doula) |
| **Respectful care descriptions and definitions** | **Definition of respectful care relevant to hospital unit** | Interviewee's definition of respectful care relevant to hospital unit. | “trying to ensure that everyone has the same opportunities to achieve, I guess in this case, a healthy outcome for their pregnancy, and doing whatever it takes to make sure that the patients are cared for in an appropriate way so that they can have the best outcomes possible.” (Obstetrician)  "Patients receiving care that is dictated by their level of clinical need and that is devoid of partiality or disparity or lack of care based on ethnicity, age maybe, and gender identification. So all patients being treated in a way that optimizes and that prioritizes their needs without penalty for being different, quote-unquote." (Obstetrician).  "every patient, no matter who they are, where they come from, what their ethnicity, culture, should be given the highest quality of care, the same amount of compassion, same amount of empathy, same understanding." (Nurse) |
|  | **Examples of respectful care (staff vs staff)** | E.g. Empathetic care, trust, shared decision making; trust building. Not limited to first person/hand experience. | "There are nice nurses, doctors. Always, "Oh, hi, it's nice to see you. I'm glad to be here." And sometimes they buy donuts or something, all food, and they inviting to us to take a plate or take a donut. And they are looking for us if they have a party, I mean, staffing party or something like that and the staff lunch. They inviting us for take a lunch with them, and it's like you're feeling like you do the right thing" (Housekeeping).  " I stand for collaboration. And I think even that-- I started the midwifery program with [obstetrician]. And what our biggest goal was was that it was an integrated collaborative model and that everyone equally collaborates and highly values…And he's like, "[interviewee], I'm not a midwife. You tell me what to do." (Midwife). |
|  | **Examples of respectful care (staff vs patients)** | Being respectful of different cultures e.g. Singing to children, having access to herbs / medications, culturally competent care. Not limited to first person/hand experience. | "I had a baby that had a diagnosis that was not compatible with life. And the parents chose-- they wanted to do hospice in the hospital. They didn't feel comfortable taking the baby home. And doctors and nurses were telling this family like, "That's the wrong choice. You need your baby at home. Don't let it die in the hospital." And I tried multiple times to be like, "This is their decision. It's not our decision." And it continued. So I ended up going to ethics, and then they were leaving the patient alone." (Nurse).  "We have patients that struggle with homelessness that come and seek care at our hospital. And we've seen them come in, and they say, "Oh, I think I'm in labor." And you're thinking maybe they're preterm labor, and we're concerned for them, but the reality is that they don't have access to shower and toileting, and we're one of the ways that they get access to shower and toileting, right? And so they're really coming in because, unfortunately, they're constipated, and they want access to toileting and a clean shower, right? And we want to be mindful that we are honoring what they need in their pregnancy but also not dismissing what could be a real clinical concern by saying, "Oh, she just needs access to toileting," right? We want to ensure that we are treating every clinical complaint and concern the way that it needs to be evaluated and then not judging. If that's what they needed, that's what they needed. It's part of the services that we provide, right?" (Nurse).  "I try to just have a conversation with people. So I think when that nurse told me, "Do you know why she's here?" X, Y, and Z, for that patient who's incarcerated, I think I just, right away, said, "I don't think that really impacts how I'm going to provide care." (Obstetrician) |
| **Assessment metrics** | **Assessment / metrics of culture or respectful care** | Specific markers/measures e.g. Number of cases/percentages/safe reports/friends and family test; quality improvement dashboard. | "our numbers themselves look good in terms of we're hitting all the metrics, all the targets, but we're still having these outcomes where patients are saying they don't want to deliver with us again or that they've had a traumatic experience or they're unhappy with their care in various ways. And so to me, that says, well, then we haven't achieved equity yet because people are still leaving the hospital and we haven't optimized their outcome. So we haven't really then achieved quality or equity. We've only achieved, quote-unquote, "safety," but we haven't achieved the psychological safety, only the physical safety." (Nurse)  "Well, patient safety, there's perinatal core metrics that you can evaluate. That's severe maternal morbidity, maternal mortality, unexpected newborn complications, your normal spontaneous term vaginal delivery rates. You're avoiding early elective deliveries. You probably know that, but perinatal core measure one, PC-O2 is the NTSV rate, episiotomy rates, all of which we excel at in our program. So patient safety is really outcome-driven. What are the outcomes across the spectrum of care?" (Obstetrician) |
| **Non-respectful care** | **Characteristics influencing non-respectful care** | Characteristics that influence non-respectful care e.g., race, ethnicity, cultural or religious practices, age, weight, mental health history, substance use, or social economic status. | "So for substance abuse, there's moms that are still using and then come visit their baby, and they fall asleep. And it's difficult to take care of because it is a safety risk, but that's only maybe one in five. The other four are in a treatment program. They're doing everything they can to change their life direction, and they want to be there for their baby. But because of that one of five that is still using and then CPS is involved, they kind of just put all these moms of substance use in a bucket and treat them like they're not safe, and they're still using, and so I don't want them touching this baby. That's what happens." (Nurse)  "I think you would be very naive to say that that doesn't come into play in the medical field. I think because medicine has made a lot of progress but used to be a very male-dominated field, you definitely sometimes see experiences where team members respond differently to male colleagues. I think every female physician has been called nurse repeatedly. And I know that, and I have listened to my colleagues who identify as Black or Brown, have, I think, probably different experiences in the hospital and in the unit than I do." (Obstetrician)  "I've definitely seen providers react to the amount of narcotic medication that the patient is requiring since it was a very high amount. I do think that-- while they may not say it, I think there's maybe a bias towards thinking this patient may be addicted to the substance." (Anesthesiologist) |
|  | **Examples of non-respectful care (between staff)** | Staff factors include race/ethnicity, culture, age, training, sexual orientation, and seniority. Includes microaggressions, emotional and physical harassment, a specifically witnessed action or statement; as well as unprofessionalism, lack of professional courtesy, and lack of self-awareness or insight. Not limited to first person/hand experience. | Racism: “And it felt like it was a barrier because I can almost guarantee if I'd been White, blonde hair and blue-eyed, I don't think I would have had to be challenged the same way, very honestly.” (Doula)  "But older generations, White individuals, I've personally been told, "Oh, you have such a long last name," or they won't even bother to learn it because it's too long.” (Obstetrician) |
|  |  |  | Sexism: “I can't help but feel like part of this comes from the fact that most of these physicians are female. And I think that plays a role in maybe a disrespect of the authority when the order is from a woman instead of a man.” (Anesthesiologist)  “I think you would be very naive to say that that doesn't come into play in the medical field. I think because medicine has made a lot of progress but used to be a very male-dominated field, you definitely sometimes see experiences where team members respond differently to male colleagues. I think every female physician has been called nurse repeatedly.” (Obstetrician) |
|  |  |  | Hierarchical (seniority): "20 years ago, there was a lot of, "I've earned this place. You are the little one." (Midwife) |
|  |  |  | Hierarchical (specialty/profession): "It was a doctor who mistreated me. And then we ended up having to go to do this conflict resolution thing because I brought it up to the director. And it got to a place where-- at first, it was just kind of microaggressions here and there, and I was feeling manipulated at times. And also, I had trouble in meetings, like bringing my full self or just being able to feel like I was being heard, and then bringing my opinions. " (Music Therapist)  “I've had OBs who've said out of their mouth that doulas are just glorified back-rubbers or whatever." (Doula) |
|  |  |  | Staffing levels: “I've witnessed microaggressions in the sense of the provider gets angry because something is not going fast enough. And it's just like, "Whoa." They want to go back for a C-section, but the unit's busy. We don't have enough staff. And they need to get back to the office.” (Nurse) |
|  |  |  | LGBTQ+: "resident who is in a same-sex relationship was sort of not harassed, but was talked to in a way that made her feel really uncomfortable about how a nurse was treating her based on her personal life. And so we definitely, unfortunately, have some issues on our unit." (Obstetrician) |
|  | **Examples of non-respectful care (from staff towards patients)** | Including microaggressions, emotional and physical harassment, specifically witnessed action or statement, as well as unprofessionalism, lack of empathy or patience. Microaggression is defined as an action or verbal comment which makes people feel uncomfortable; microaggression is defined by the subjective experience of the victim/recipient. Not limited to first person/hand experience. | Racism: "And I've seen, with people of Black and Brown color, where they'll have these conversations at the bedside, and they talk down to these parents. But yet, if you're White or Caucasian, the tone of voice is different, and the way they treat these patients is different. And I've seen that in every hospital that I've worked at in California and across the US." (Nurse)  "My white clients never, never encounter the line of questioning about fear of your partner, and are you safe at home, and do you have-- it doesn't happen." (Doula)  "Sometimes if certain races I've heard-- again, these are older White attendings, some of whom have retired, will say, "Oh, that race or that particular last name means that they probably can't have a vaginal delivery, or it'll be really challenging, or pain control won't be good." Or they do make assumptions. I have heard that." (Obstetrician) |
|  |  |  | Hierarchical (seniority): "So the charge nurse, I talked to the charge nurse and told her about the program and what I do. And after I talked to her, she went to the client and said, "Are you sure that they can provide good support for you? Because after all, the program's free. The program is free to the client. The program's free. Are you sure? I mean, do you really trust that that doula can really do this?" So what happened is I go into the hospital. And so this has been-- this is what's been said to my client and her husband. And they're upset and agitated because if I hadn't really established a rapport with them before, it would be easy for them to question it. If you have a medical professional questioning a doula, then why would you trust that person? Like I said, unless we had that rapport." (Doula) |
|  |  |  | Hierarchical (specialty/profession): “those moms are the ones that really-- like, "I wish I knew that I could have waited. I wish I knew that I didn't have to go ahead and get induced. But the doctor said this," or, "They said that." But I feel like they're not giving them conversations where they know that this is something that they should be able to decide together, especially as long as it's not a true high-risk situation." (Doula)  " I've definitely heard labeling of male support persons as, "Well, they're just angry. They're hostile. They are identified as a threat," conveying to them, "If you continue to talk to me this way, I'll just call security." You can't remove this person from their partner's birth experience because you don't like the way they communicate with you, right? And so there's a lot of dynamics there where oftentimes when you dig into it, yeah, they may be communicating with what you perceive as anger, but if you dig into that deeper, maybe they're just scared, right, and they're fearful because they've also read the lay press, and they know that their partner is at a higher risk of mortality than others, right?" (Nurse) |
|  |  |  | LGBTQ+: "For me, if I had to-- I cared for a transgender patient, and it is something that I don't believe in. It is something that I believe; God's created us as men and women. That's my belief. But I still stepped in and was very honest, like, "I may not call you a he. It will be very hard for me. But it does not mean that-- I don't disrespect you." Because I felt like if I went in there, and I just was not honest and said she, then they're going to start to build a bias, like, "They don't care. They don't respect me. They don't do this." And I wanted them to hear from me, like, "I respect you as a person, but it is going to be hard for me. This isn't something that just comes natural to me."" (Nurse) |
|  |  |  | Abortion: "For example, I will use the example, a long time ago, probably like eight years ago, we had a patient who had a nonviable baby. And again, my beliefs, I don't believe in abortion, but the baby was not going to be viable at life. And she was probably 24 weeks and decided she wanted to deliver. And I remember still-- maybe it was more. I was still probably a baby new nurse. And a few nurses had said, "Oh, I don't want to care for her. Basically, it's an abortion." (Nurse) |
|  |  |  | Ageism: “[the delivery] was very traumatic. And it was just based on a presumption that the girl's mother didn't look old enough to be her mother. So there weren't any marks on the teenager. There was never any concern about her being physically abused. She never stated that she had been sexually assaulted or physically abused. It was just based on the fact that the biological mother, quote-unquote, "looked too young to have a 19year-old daughter." (Obsetrician) |
|  |  |  | Drug and substance misuse: "So for substance abuse, there's moms that are still using and then come visit their baby, and they fall asleep. And it's difficult to take care of because it is a safety risk, but that's only maybe one in five. The other four are in a treatment program. They're doing everything they can to change their life direction, and they want to be there for their baby. But because of that one of five that is still using and then CPS is involved, they kind of just put all these moms of substance use in a bucket and treat them like they're not safe, and they're still using, and so I don't want them touching this baby. That's what happens." (Nurse) |
|  |  |  | Weight/obesity: "Unfortunately, a big one on my unit is comments pertaining to a patient's body mass index, particularly if they're obese or morbidly obese. I have just heard some comments that are just not clinical. And luckily, they're not in front of the patients, but I think they could be upsetting to staff or to the patient if they heard that." (Anesthesiologist) |
|  |  |  | Staffing Levels: "I've worked other units where the parents are like, "I haven't had an update in the doctor in like three days." And then you talk to the doctor, and they're like, "Yeah, I'm busy." So that also varies by hospital too." (Nurse) |
|  |  |  | Socioeconomic status/affluence: "But I had one client who showed up, again, low socioeconomic, all these things. And she told the nurse she had a doula. And she said, "You have a doula? How do you have a doula?" So the client was-- for her to make that comment, it just wasn't appropriate. Even if she thought that, that was something she could have kept to herself. So that idea of, how did you get one?" (Doula) |
|  |  |  | Stigmatized medical illness (e.g. HIV, hepatitis B): "Mental health…people talk about, "This mom has depression or postpartum depression," … And I'm like, "Why are you talking here in the room, and the mom can hear you talking about her?”... It's not like you need to tell people's business in a room full of other people." (Music Therapist) |
|  |  |  | Language barrier: “There was a mom, that she-- oh gosh, what was the language she spoke? Anyway, she was from the Middle East. And I worked with this mom because she wanted me to record prayers for her baby…and the staff, I remember one commented that-- one of the nurses said, "Oh, this mom is constantly just shaking the baby and singing for the baby and praying over the baby. I think the baby is overstimulated." And for a minute, I thought that that was the case because they were so convincing, right? But then another nurse who was really more observant, she's like, "Hey, I think that that actually works for this baby." (Music Therapist) |
|  |  |  | Disability (physical/mental/behavioral) e.g. learning difficulties, ADHD, wheelchair bound: "I mean, I was at another hospital where the psychiatry department was essentially useless. They were basically there to say, "Do we 5150 of them or not?" Right? And if you're not 5150, they were just useless. " (Obstetrician) |
|  | **Examples of non-respectful care (from patients towards staff)** | From patients, directed towards a staff member. Including microaggressions, emotional and physical harassment, specifically witnessed action or statement, as well as unprofessionalism, lack of empathy or patience. Microaggression is defined as an action or verbal comment which makes people feel uncomfortable; microaggression is defined by the subjective experience of the victim/recipient. Not limited to first person/hand experience. | "I had a nurse that was from India, and the patient says, "I don't like how she smells, and I need a new nurse."" (Nurse) |
|  | **Impact of non-respectful care on patient treatment and care** | Reaction, feeling secondary to example or incident. | "What makes me sad about birth plans is that the patient feels like they have to say, 'I want to make sure that I don't experience harm.'" That was a bullet point on a birth plan. Gosh, for patients to feel like that's important to express means that they feel that there might be someone intending to cause harm, right, or scared of their safety in a hospital, which is great to express to keep it up there for everyone to realize, "Wow, we're not doing a great job helping people feel safe."" (Midwife)  "I feel that the patient even themselves may feel a little hesitant when they first step up because equity is such a huge thing. Like, "Is my voice going to be heard? Am I going to be told one thing? Am I going to be believed if something happens?"", "t was like your experience with C-sections and stuff. And it was very disheartening - I was telling my husband - to hear how every single person who was speaking felt that they-- or nurse didn't hear them, that they didn't matter. Their husband didn't matter. And it was just heartbreaking " (Nurse). |
|  | **Impact of non-respectful care on staff treatment** | Reaction, feeling secondary to example or incident. | "I recently overheard someone say, "I don't want to voice myself out of the job.”" (Midwife) |
| **Strategies impacting respectful care** | **Onboarding processes / information** | Onboarding training / tips for new hire. | "When I talk to new staff, I'm telling them that, "I want people who want to be part of making it different, who want to be part of making that change. And if you don't, that's okay as long as you recognize it in yourself and you know that it's going to move forward. And so if you're a person that's going to be dragging it back, then you're going to be on a different pathway with someone like me." Those are hard conversations to have." (Nurse) |
|  | **Training and education** | Can be positive or negative examples. E.g. Nurse training, including implicit bias training. | " I know there are opportunities, but in all honesty, I think because we're the county and every minute that we work is so highly scrutinized, in general, we don't really get faculty development. There isn't much time allowed for us to do a lot of these things, unfortunately." (Obstetrician)  "Everyone at our institution has to take those online forums or whatever where you kind of click through the modules. You know what I mean? But that just isn't sufficient enough, I think. We had a lot of really good luck in the past doing similar sort of small group discussions, and having these-- for example, patients coming and discussing their experiences when it came to perinatal loss and abortion." (Obstetrician) |
|  | **Leadership strategies** | Team leader led initiative, action based, examples of open-door policy | “I caught a significant mess of a patient who'd had an eclamptic seizure during an attempted home birth - was to reach out to the home birth community to set up quarterly meetings led by a midwife, not led by a doctor, to basically lower the barrier to entry to say to everybody, "You can come here no matter what. We're not going to steal your patient. We're not going to badmouth you. We're not going to say, 'You shouldn't have sent her in." (Obstetrician)  " If we have good leadership, if we have good role models and people who are modeling equity for all, and ensuring that everyone has equity. I also think a culture where everyone is on the same page, everyone is educated. And I also think it's important to hire diversity within your team." (Obstetrician)  " I think our managers and our leaders, at least from my perspective, have an open-door policy of like, "If there's anything going on, we want to hear from you, and you'd be protected and safe." That's all I kind of know...during the time of transition where she was expanding to more assistant program directors, I reached out and said, "Hey, if there's any space for a midwife to be part of this, what would that look like?" And she's like, "No, no, no. This is what we want to represent because it shows how much our program honors what the breadth of midwifery would bring to obstetrics and, professionally, midwifery and obstetrics together." (Midwife) |
|  | **Communication strategies** | How communication is used to achieve respectful care e.g. Multimedia, handouts. How information is communicated to patients or staff. Lack of patient complaints, how to stand up for oneself; negative descriptions of communication, intentional feedback to influence culture and behavior. | "when you're rounding, you should have the families there in the round. There's a lot of NICUs that are doing parent-led rounds where like, "My baby is 32 weeks and is doing great on feeding," and let the parents be the first person that talks. Why is it the nurse or the MD? They're the patient. They should know what their plan is. It's their plan. [laughter] So one of the most effective things for family-centered care is rounding, is inclusion of the families. And so when they're not available, that's where you use your video conferencing, whatever that is, and then they can virtual into those meetings." (Nurse)  "The minute it's happening, they do talk about it to each other. They do try to figure out what they can do so it's not going to happen again and try to figure out what steps they should take so that it doesn't happen anymore. Conquering the problem, basically. " (Front desk) |
|  | **Encourage Self Awareness** | How a system works for an individual or realizing inequities are an issue; improving respectful care through self-improvement; understanding risk/benefit ratio as a provider, including accountability (to staff, to leadership, to patients) | "One of the things is self-awareness and having awareness of external issues and the challenges other people face while still having an internal locus of control. So people who are self-aware enough to realize their strengths and weaknesses, empathetic enough to realize the needs of others, and by having an internal locus of control, they feel, "I can help take actions to create the change." (Obstetrician)  “It requires so much self-awareness. And in turn, the more self-awareness you have, the more you can tap into empathy. And so teaching self-awareness and empathy so that way you can drive positive culture as a leader is really difficult because sometimes when you're trying to create self-awareness for an individual, it requires disciplinary action, and that is hard as a leader." (Nurse) |
|  | **Hospital Strategies to improve respectful care and culture** | Division or department-led initiative. E.g. commitment to equitable care documents; policies/protocols informed by data, e.g. Quality improvement meetings, health equity tasks; safe reports, quality reviews | "We have this larger goal. It's great. It's heavily influenced by Joint Commission and our California work. And we've been relatively successful in the outcome measure, but all the processes that need to go into that are still being developed. So we have a lot of work to do within the equity goals." (Nurse)  "The number one thing is standardization of care. I feel like when you have standardized care, when you have policies and protocols in place that everyone is following, then that really reduces that equity gap." (Nurse) |
|  | **Identifying social needs of patients** | E.g. Partnering with communities, navigating social services | "And that's just the beginning, right, because then there's a whole place for doulas and community health workers and all these other players to interact in the hospital care environment so that way, at the end of the day, when the patients leave our care, even if it's only for three or four days, that we have set them up to have better health outcomes. And when I say that, one of the things we've been working on in our mission and vision is to really say, "How do we set families up to break generational healthcare patterns or generational negative healthcare patterns so that way we can set them on a trajectory to have a positive healthcare pattern emerging?" We can't guarantee them a completely different health outcome, but we can potentially create an experience where we change that trajectory in healthcare patterns." (Nurse)  "isn't what they chose, but what brought them in here is what is impacting the health now. Or their chronic health has now created this because of their socioeconomic status. " (Midwife)  "I think one of the biggest barriers is hospitals have been on the left, and then public health and community on the right, and they haven't been talking to each other. So if it was ideal world and everything was perfect when you were in the hospital but then you don't provide the resources for them when they're back in the community, you're still going to have readmissions. You're still going to have mortalities. So I feel how this is going to work is when the hospitals and the communities work together." (Nurse) |
| **Challenges and Barriers impacting respectful care** | **Challenges / barriers to achieve respectful care and outcomes on the hospital unit** | Includes changing population that is being served e.g. religion, ethnicity, e.g. Trying to start initiatives/making a change but facing bureaucratic challenges to execute this; resources e.g. Inadequate staff, also lack of trust | Financial resources: “So it's hard to provide equity for patients who just don't have the financial resources to be able to support what their needs are. So whether it's a patient who needs to see a nutritionist because her diabetes is poorly controlled and her health insurance doesn't provide for that, where the level of care that she gets from the nutritionist is subpar to the person who is wealthy and can have access to a well-funded or well-trained nutritionist and is able to meet all of her nutritional needs through a personal chef. So equity would be hard to achieve for those patients in those two separate situations. I think most of it comes down to finances.” (Obstetrician) |
|  |  |  | Infrastructural resources: “And in group prenatal care in Centering Pregnancy, they give you this, like, "This is how the model works, and it's only for low-risk pregnancies." And so we started the way that we were guided to because that's how the model works. But now we're speaking up, saying, "Okay. Wait, this model isn't equitable yet because we're not able to offer it to our high-risk mothers." We're not able to offer it. We just opened a Spanish-speaking group. So now we're trying to reach our cultural Spanish-speaking group and trying to identify other groups where group care really is a great way to find a subset, so not necessarily isolate or call them out, but actually promote what they're experiencing. So we're looking at diabetes, LGBTQ. So that's one way. I'm trying to improve that access.” (Midwife) |
|  |  |  | Staffing / workforce capacity:  “They shut the unit for a couple of years, waited, then reopened it with a whole new crew. It is very hard to change culture. It is actually easier to change people because there's so much you need to” (Obstetrician)  “I think it's very important because most of the patients we serve are Spanish-speaking. So if you have a native Spanish speaker or someone who understands the culture and the background, they can connect better with the patients and provide better care, I think because they understand where they're coming from culturally. And then I think that really improves health literacy.” (Obstetrician) |
|  |  |  | Communication: “I just don't think that there's enough education or discussion about it. And I think anybody who discusses about it becomes offended with it.” (Nurse) |
|  |  |  | Biases:  “The reason was that the patient had suicidal ideation about carrying this pregnancy to term, and her physician had agreed under those terms to allow her to terminate this fetus that was beyond our usual cutoff for which we do that. So I heard about many providers being upset about the way in which this abortion care was handled in the setting of-- and I think that brings in some issues of maternal autonomy since-- I mean, my principle, at least, is that maternal well-being is more important than fetal well-being before the fetus is born. Because if Mom is not healthy, then the baby cannot be healthy. So that was definitely a tricky-- this is just one example of a tricky situation in which people had, I think, their own views of what abortion means and what type of abortion is acceptable. And some staff were unwilling to accept the care situation." (Anesthesiologist)  “I mean, you can't educate people enough, and you can't just bring the awareness because I think a lot of these things are the biases, and the actions, I think, most often are unintended or even unrecognized, or people don't realize it was even a piece that's considered something that is a bias or inequity or something that is happening. So the more education there is, the more awareness there is around it” (Midwife) |
|  |  |  | Socioeconomic determinants of health: “I would say probably the biggest are the social determinants of health. When I meet my patients for the first time on labor and delivery and clinic, there are definitely many more barriers than if I was taking care of a population that was from a higher socioeconomic community, whether that's transportation, food, education, you name it. And we all know that those have a lot of impacts on our health.” (Obstetrician) |
|  | **Challenges of responding to non-respectful care** | Examples of how people find it difficult to respond to non-respectful care. | “And I think people get tired. You get really exhausted calling out bad behavior.” (Nurse) |
| **Wellbeing** | **Importance of self-care as staff member** | Reflections on why self-care is needed, coping strategies. | "We really do practice self-care, and I particularly practice debriefing with particularly hard births because I can't sit in that. Because if I sit in it, it'll just become even more overwhelming. And there's been a lot of times where I'm able to express how flustered and angry I am as well." (Doula)  "Taking care of the staff there at the bedside, also their mental health, I think, is really important, and allowing them time for that. And that really depends on where you work and the resources available." (Nurse) |
|  | **Examples of self-care by staff members** | What participants are doing for selfcare. | "And yeah, the things I do to kind of help relieve the challenges often are things like meditation or talking things out or journaling or yeah, kind of using those venues to release some of that either sadness or stress or frustration." (Obstetrician)  "I'm someone that really needs to talk about it. And so I'm lucky that I have a few colleagues that I really trust and value their opinion, and they're great listeners. And these are colleagues that I've had since med school. Some are residents. And talking about it is very helpful. As I kind of got through my residency and after having my daughter, I also tried to keep better boundaries of work. I do my work at work, and I really try not to log in and do patient care at home. And so I think that has also helped." (Obstetrician) |
|  | **Challenges of caring for oneself as hospital staff member** | Examples of challenges of caring for oneself as hospital staff member. | "I was taking care of triplets, and they passed away on my shift. All triplets did. And you just have to keep going. And I'm like, "That would have been much better and less for burnout if I had had time to go into a room and just sit for a few minutes," versus like, "Okay, next patient, next thing." Taking care of the staff there at the bedside, also their mental health, I think, is really important, and allowing them time for that. And that really depends on where you work and the resources available." (Nurse)  "I do remember them talking about traumatic experiences. Because of my role, I just hear it, and I am so amazed that these girls are able to go through what they go through and still manage to come back. Some days, even the next day, or still continue to finish their 12-hour shifts after what they go through. The department really has nothing for these nurses, or for anybody, really. I don't see any help. There's no help for what they go through when they do go through some trauma and experience what they do. So I don't see anything there. There's nothing there to help them." (Front desk) |
|  | **Burnout** | Emotional, physical, mental sequelae e.g. Burden of taking responsibility in advocating for respectful care. | "I feel like, in the past, you just internalize it, but then that is not good for anyone. That leads to long-term PTSD and then burnout, which is why I left the hospital setting." (Nurse)  “when I went to my doula training six years ago, there was about 25 people in the room, and only 3 of us are still doing the work, right? So it's like, yeah, burnout is real. All these things are real, right? But I feel like it's like, you really have to take care of yourself before you could even attempt to go take care of anyone else. You got to make sure you're good. And that's how I continue.” (Doula) |
|  | **Guilt** | Blaming oneself e.g. For not speaking up, having bias/prejudging a patient. | "And I have to say, I am guilty of that. And it was eye-opening because I had one patient, and that was the situation. She was a larger woman. And those are the first things you're thinking. And so I walk into the room to see her, and she's just crying. She's sitting in the bed just crying. I'm like, "Oh, my God. What's wrong? Are you okay? Are you safe? What's happening?" She says, "I am so scared that I'm going to lose my baby." And my heart broke for her. I'm like, "Oh, my God. She's a woman and she's a mother, or mother-to-be. And she's afraid." And to prejudge her made me so-- I was so disappointed in myself. I'm like, "Oh, my God. She just needs help and support and someone to talk to her and someone to listen to her." So I mean, I will say I have been guilty of that. And it's a terrible, terrible thing. But take the time to get to know somebody, and it humbles you." (Nurse) |
