# Supplementary material for: Unit culture and respectful care in obstetric care settings: a qualitative concept elicitation study
Source: Front Glob Womens Health. 2026 Aug 10;7:1833740. doi: 10.3389/fgwh.2026.1833740 (PMC13502073; doi:10.3389/fgwh.2026.1833740)
Supplement: Supplementary file 5 [file Supplementaryfile5.docx]

Supplement 5. Definition of unit culture and respectful care

| **Theme** | **Summary of analysis** | **Common concepts** (number and % of all participants who articulated these concepts) | **Illustrative quotes** |
| --- | --- | --- | --- |
| **Definition of unit culture** | 15 participants reported on the definition of unit culture.  Common attributes were interpersonal relationships, teamwork, leadership, interactions between people, shared priorities and goals, attitudes, and the “feeling” of the unit. | Patient‑centered care, safety, and outcomes orientation (9/15; 60%)  Describes shared mission/goals/ philosophy (8/15; 53%)  Leadership/ accountability structure (6/15; 40%)  Teamwork and collaboration across roles (8/15; 53%)  Showing respect, kindness, dignity, caring in actions (13/15; 87%)  Communication and how people speak to each other (5/15; 33%)  The atmosphere/feel/ morale of the unit (10/15; 67%)  Mention of diversity, inclusion, gender, race, identity (6/15; 40%) | "It's an energy, right? The energy that the unit has. Or if I were to describe it, unit culture-- I never even thought about it like that. But yeah, I would say a way of being, different ways they treat people, how they talk to people, how it feels, things like that." (Doula)  "culture is really around the interpersonal treatment, how we treat our-- how do I say that? The interpersonal dynamic within the team itself. So there's that component of it, how we show up to work every day, how we interact with each other as coworkers, colleagues, peers, and how we convey mutual respect to each other within the team. And then as a result of those team dynamics, it contributes to the morale of the unit. So for me, the culture is, how do we drive a morale that is mutually inclusive, conveys respect, kindness? And then as a result of that particular team dynamic, then we have the culture that is involved with how we communicate, convey respect, kindness, and care to the patients…really hard to define in the healthcare setting because it encompasses so many things, teamwork, communication, collaboration, like I said, kindness. " (Nurse)  "it would be the kind of shared mission, drive, and philosophy of the unit and how people treat and interact with each other." (Obstetrician).  "unit culture is how the unit works collaboratively for successful outcomes. Everyone engaged in not only the medical care, but patient satisfaction, patient experience, and patient safety. And culture is part of everyone getting along, being collaborative, no shaming" (Obstetrician).  "It would be just overall the both implicit and explicit component of priorities. And with a high-value culture, we placed high priorities on all the people working on the unit, all the people we care for. So it would be the character, the goals, the communication style...I would describe unit culture as something that is the sort of underlying goals for how we treat our patients and how we treat one another and just sort of our way of approaching the work that we do, and that involves how we treat each other and how we treat our patients. So that's what I would say is our sort of unit culture. And it's sort of our attitudes about working together as teams and things like that. So just generally, I would say, the approach to patient care of our unit and how we prioritize how we treat people." (Obstetrician)  "it's the feeling when you walk into a unit, you know right away from conversations between the nurses, interactions between nursing and physicians, just listening to how they speak to the patients, what that culture is like. Is it cohesive? Is it compassionate? Is it volatile? Is it toxic? Just walking onto the unit. So for lack of a better way of putting it, it's the feel of a unit to me. It's the way they function, the way they interact with each other, the way they interact with families and patients" (Nurse) |
| **Definition of respectful care** | 17 participants reported on the definition of respectful care.  Common attributes were equal, bias-free care based on clinical need, respect and kindness, individualized care with patient voice, teamwork and consistent standards. Common terms used to describe this phenomenon were care, equity, patients, culture, everyone, people. | Bias-free, need or diagnosis-driven care (14/17; 82%)  Individualized, person-centered care and accommodations (14/17; 82%)  Respect, dignity, kindness, and humanization (12/17; 71%)  Team-based and family culture (9/17; 53%)  Equitable care (8/17 47%)  Equal standard of care regardless of identity or status (6/17; 35%)  Protocols or checklists to ensure consistent minimum standards (4/17; 24%)  Interprofessional culture (6/17; 35%) | "Patients receiving care that is dictated by their level of clinical need and that is devoid of partiality or disparity or lack of care based on ethnicity, age maybe, and gender identification. So all patients being treated in a way that optimizes and that prioritizes their needs without penalty for being different, quote-unquote." (Obstetrician).  "Just treating patients, others with dignity. And I want to say fairness, but it's such a-- I don't know. Yeah, fairness, no matter where they came from, no matter who they are, if they don't speak our language. Or it's just really providing the same level of care, the same level of respect and being attentive." "So yeah, I think it's just having respect, treating people as you would like to be treated. " (Music Therapist)  "every patient, no matter who they are, where they come from, what their ethnicity, culture, should be given the highest quality of care, the same amount of compassion, same amount of empathy, same understanding." (Nurse)  “exercising empathy to understand each individual's needs so that way we can then achieve the best possible outcome” (Nurse)  "Basically, that everyone receives the exact same access and treatment to resources, personnel, and opportunity for each of those." (Midwife)  “trying to ensure that everyone has the same opportunities to achieve, I guess in this case, a healthy outcome for their pregnancy, and doing whatever it takes to make sure that the patients are cared for in an appropriate way so that they can have the best outcomes possible.” (Obstetrician)  “Everyone's treated the same. There's not this bias when you enter a room. In a perfect unicorn world, that's how it would be. Every patient would be treated with respect, would be heard, every family member. It would be a very cohesive type of team, including the patient and the family.” (Nurse) |
